# Esophageal Dieulafoy Lesion: A Rare and Potentially Fatal Entity

**DOI:** 10.1155/2021/8896489

**Published:** 2021-03-04

**Authors:** Eoghan P Burke

**Affiliations:** Royal College of Surgeons Ireland, Dublin, Ireland

## Abstract

A 35-year-old gentleman presented acutely to the emergency department with large volume haematemesis and melena. Following adequate initial resuscitation, the patient underwent emergency upper gastrointestinal endoscopy which revealed a dilated tortuous submucosal vessel which was actively bleeding at the midpoint of the esophagus. This was consistent with a Dieulafoy lesion. However, its position in the midpoint of the esophagus is rare. Our patient recovered well following intervention.

## 1. Introduction

Dieulafoy lesions are a rare but important cause of massive gastrointestinal (GI) bleeding. They account for less than 2% of gastrointestinal bleeds but have a significant risk of morbidity and mortality secondary to delayed diagnosis and treatment [1]. The underlying pathophysiology of these lesions is poorly understood. They are classified as dilated tortuous histologically normal submucosal arteries [2]. They classically occur in the stomach on the lesser curvature but have been reported in the colon, duodenum, and rarely in the esophagus as in our report [3]. Patients typically present with acute onset large volume haematemesis and/or melena. Unlike bleeding from a Mallory-Weiss tear, bleeding from a Dieulafoy lesion is not typically associated with preceding retching or vomiting [4]. Diagnosis usually involves endoscopy although there is a growing role for angiography particularly if suspicion remains following a normal endoscopic evaluation. Endoscopy has the added advantage of being therapeutic as well as diagnostic [5].

## 2. Case Presentation

**Figure 1:**
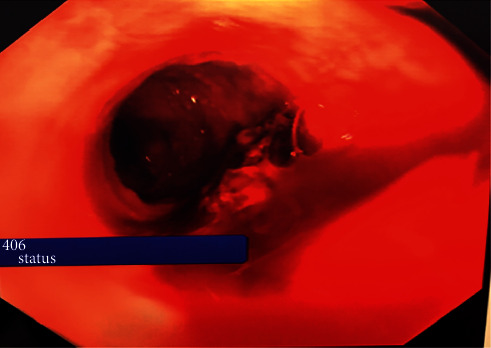
EGD image of active bleeding from a visible dilated tortuous vessel at the midpoint of the esophagus. His bleeding was controlled using endoclips and haemostatic powder (Figure 2).

A 35-year-old gentleman presented acutely to the emergency department with haematemesis and melena. He had no past medical or surgical history and was not taking any regular medications. He worked as a secondary school teacher. Of note, he had no risk factors or diagnosis of liver disease, cirrhosis, or portal hypertension. The haematemesis and melena occurred acutely with no preceding vomiting. He had been attending a choir rehearsal with his class of students when the bleeding occurred. On clinical examination, he was noted to be of normal BMI. He was hypotensive and tachycardic in the emergency department, and laboratory investigations were significant for a haemoglobin of 8 g/dl. The massive transfusion protocol was activated, and he was prepared for emergency endoscopy. At esophagogastroduodenoscopy, he was found to be actively bleeding from a visible dilated tortuous vessel at the midpoint of the esophagus. The bleeding was pulsatile in nature. The findings were consistent with a Dieulafoy lesion (Figure 1).

He remained stable with no further bleeds. The patient was counselled on the need for further evaluation to determine whether a definitive procedure was required to excise this vessel completely given its high risk of rebleeding. However, he refused further investigations/interventions. He was discharged home well.

## 3. Discussion

Dieulafoy's lesion was initially described by Gallard in 1884 as “military aneurysms of the stomach.” However, it became synonymous with the French surgeon Dr Georges Dieulafoy in 1898 when he more accurately characterised them during his investigation of fatal upper gastrointestinal haemorrhage in three young asymptomatic men [6].

These lesions are classically found in the stomach predominantly on the lesser curvature. The precise reason for this remains controversial. Some have attributed it to the fact that the submucosal blood supply in this region of the stomach arises directly from the right and left gastric arteries which run adjacent to the lesser curvature suspended in the hepatogastric ligament and so do not have to transit a significant distance prior to reaching their end-organ [7]. However up to 1/3 of Dieulafoy lesions may occur outside the stomach, predominantly in the duodenum and colon. The aetiology for these lesions, at sites other than the lesser curvature of the stomach, remains unclear [8].

The underlying pathophysiology for the development of these lesions is poorly understood. These lesions are histologically normal, when analysed in resected specimens, but maintain a larger calibre in comparison with adjacent blood vessels. It is well documented that a normal artery of the gastrointestinal tract will narrow as it traverses the wall of the organ it is supplying [9]. This, however, is not the case in Dieulafoys lesions as these vessels maintain the same patency throughout their course. As these vessels remain larger in diameter within the submucosa, they may cause damage to the overlying mucosa secondary to forceful pulsation. Should the overlying mucosa breakdown these vessels are then exposed and subject to trauma and thus haemorrhage, often without any proceeding symptoms [10].

These lesions are reported as being rare in the esophagus [11]. A literature review performed on PubMed using the following search string: ((Dieulafoy Lesion) AND (Dieulafoy's Lesion)) AND (Oesophagus OR esophagus) yielded 32 results. On review of the abstracts of these reports, 10 were found to pertained to Dieulafoy's lesions in the oesophagus presenting with upper GI bleeding and were published in the English language with abstracts available.  Table 1 reviews these previous reports. It remains unclear, however, whether this is due to under diagnosis. These vascular abnormalities can present with massive upper gastrointestinal haemorrhage and can be fatal if haemostasis cannot be obtained. If identified they can often be managed effectively with standard endoscopic techniques.

These lesions are under recognized and should be considered in all cases of acute GI bleeding.

## Figures and Tables

**Figure 2:**
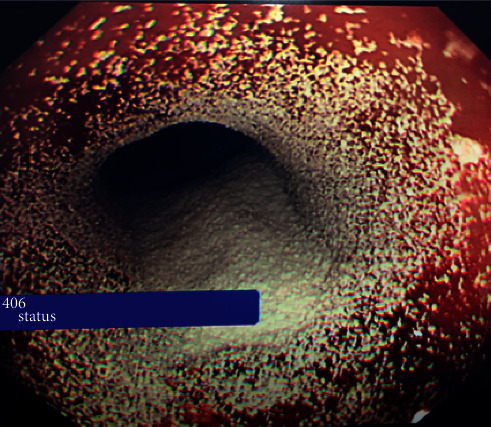
EGD image postsuccessful haemostasis using dual therapy of endoclips and haemostatic powder.

**Table 1:** Summary of previously published case reports and case series pertaining to Dieulafoy lesions in the oesophagus.

Publication	Demographics	Presentation	Site of lesion	Treatment and outcome
Inayat, F., Ullah, W., Hussain, Q. and Hurairah, A., 2017. Dieulafoy's lesion of the oesophagus: a case series and literature review. Case Reports, 2017, p. bcr2016218100.	3 patients			
	53 years old caucasian male.	1 week history of melena	Distal oesophagus	Adrenaline injection and haemoclips
	68 years old Caucasian male	Admitted following RTA, on day 2 of admission developed fresh rectal bleeding	Distal oesophagus	Haemoclips
	80 years old Caucasian male	2-day history of haematemesis	Distal oesophagus	All three patients recovered well and were discharged home with no further intervention

Nemakayala, D.R., rai, M.P., Yam, J.L. and Laird-Fick, H., 2018. Dieulafoy's lesion in the oesophagus: A rare cause of upper gastrointestinal Bleeding. Case Reports, 2018, pp. bcr-2017.	55 years old Caucasian male.	3-day history of melena and a presyncopal episode	Distal oesophagus	Adrenaline injection and gold probe cautery
				Discharged home with no further intervention

Ertekin, C., Barbaros, U., Taviloglu, K., Guloglu, R. and Kasoglu, A., 2002. Dieulafoy's lesion of esophagus. Surgical Endoscopy And Other Interventional Techniques, 16 [1], pp. 219–219.	25-year-old female	Haematemesis and melena	Distal oesophagus	Band ligation
				Discharged home with no further intervention

El Hajj, I.I., Malik, S. and McGrath, K.M., 2010. Endoscopic clipping of Dieulafoy's lesion in the upper esophagus. Digestive and Liver Disease, 42 [2], pp. 155–156.	61-year-old male	Haematemesis.	Upper oesophagus	Haemoclip
				Discharged home with no further intervention

Benatta, M.A. and Grimaud, J.C., 2017. Band ligation for a gastroesophageal junction Dieulafoy's lesion. The Pan African medical journal, 26.	24-year-old male	Haematemesis	OGJ	Band ligation
				Discharged home with no further intervention

Yoshida, T., Adachi, K., Tanioka, Y., Sasaki, T., Ono, S., Hanada, H., Esaki, T., Yagawa, T., Takeo, S., Saiki, Y. and Harada, T., 2004. Dieulafoy's lesion of the esophagus correctly diagnosed and successfully treated by the endoscopic injection of N-butyl-2-cyanoacrylate. Endoscopy, 36(02), pp. 183–185.	74-year-old male	Haematemesis and melena	Distal oesophagus	Injected with N-butyl-2-cyanoacrylate
				Discharged home with no further intervention

Ho, K.M., 2004. Use of Sengstaken-Blakemore tube to stop massive upper gastrointestinal bleeding from Dieulafoy's lesion in the lower oesophagus. Anaesthesia and intensive care, 32 [5], pp. 711–714.	71-year-old male	Haematemesis	Distal oesophagus	Adrenaline injection and bipolar diathermy
				Further bleed controlled with Sengstaken–Blakemore tube
				Repeat OGD did not identify a bleeding point
				Discharged home with no further intervention

Malliaras, G.P., carollo, A. and Bogen, G., 2016. Esophageal Dieulafoy's lesion: An exceedingly rare cause of massive upper GI bleeding. Journal of surgical case reports, 2016 [6], p.rjw074.	55-year-old male	Haematemesis	Distal oesophagus	Adrenaline and haemoclip placement
				Discharged home with no further intervention

Thimmapuram, J., Shah, M. and Srour, J., 2011. Esophageal Dieulafoy lesion: An unusual cause of GI bleeding. Gastrointestinal endoscopy (Print), 73 [5], pp. 1055–1056.	55-year-old male	Haematemesis	Mid oesophagus	Adrenaline injection and haemoclip application
				Discharged home with no further intervention

Abraham, P., Mukerji, S.S., Desai, D.C. And Joshi, A.G., 2004. Dieulafoy lesion in mid-esophagus with esophageal varices. Indian journal of gastroenterology: official journal of the Indian Society of Gastroenterology, 23 [6], pp. 220–221.	54-year-old male	Haematemesis	Distal oesophagus	Band ligation

## Data Availability

The data used to support the findings of this study are included within the article.
